# Supplementary material for: Pelvic floor exercise: Awareness, knowledge, beliefs and practices among pregnant women in a Ghanaian setting
Source: PLoS One. 2026 Apr 13;21(4):e0347199. doi: 10.1371/journal.pone.0347199 (PMC13075718; doi:10.1371/journal.pone.0347199)
Supplement: Attachment — Submitted filename: RESPONSE TO REVIEWERS.docx [file pone.0347199.s002.docx]

P.O. Box ST, 138

Stadium-Kumasi

16^th^ February, 2026

The Academic Editor

PLOS ONE

Dear Editor,

**LETTER OF RESPONSE TO THE EDITOR’S AND REVIEWERS’ COMMENT ON THE OF MANUSCRIPT ID:** **PONE-D-25-46176** (**PELVIC FLOOR EXERCISE: AWARENESS, KNOWLEDGE, BELIEFS AND PRACTICES AMONG PREGNANT WOMEN IN A GHANAIAN SETTING)**

I write in response to your request that I revise the manuscript of ID **PONE-ID:25-46176.**

I appreciate the constructive feedback provided by the reviewers and the opportunity to revise and resubmit the manuscript.

I have carefully addressed all comments raised by you and the reviewers. The detailed point-by-point responses are provided in the table below, with corresponding changes highlighted in the revised manuscript.

I believe the revisions have strengthened this manuscript and thus meets your journal standards and consideration

Thank you.

Yours sincerely,

**Bright Anneh Awaitey**

| **COMMENTS** | **RESPONSE** | **Line location** |
| --- | --- | --- |
| **EDITOR** |  |  |
| Marked up copy of manuscripts with highlighted change | Uploaded | N/A |
| An unmarked version of revised paper without track changes | uploaded | N/A |
| manuscript meets PLOS ONE's style requirements, including those for file naming | Addressed using PLOS ONE style templates as guide | N/A |
| Data Availability Statement i | Uploaded as Supporting Information file | N/A |
| **REVIEWER 1** |  |  |
| Clarification on Sample Selection:  Elaborate on whether pregnant ladies with BOH, complaints of UTI, neurological deficit, or any pelvic related problems were excluded from the study. | Pregnant women were not excluded on pelvic floor disorders, UTI symptoms, or other pelvic-related problems. | Line 136 -138 |
| Discussion of results:  a brief explanation of why only “Beliefs of 24 participants in section 3 towards PFME” were projected, when 47 subjects actually had knowledge about PFME. | The stated 24 participants was an error. This has been deleted | = |
| Implication for Practice:  Expand a little further on how this study may be translated into clinical or public health practice. A short paragraph in the discussion would improve the practical value of the paper | This has been addressed as part of the discussions and a short paragraph has been introduced as well | Line 245 to 331 & 332 to 340 |
| **REVIEWER 2** |  |  |
| Study design and sampling- Justify the choice of hospitals, present an a priori sample size estimation, and provide a sample recruitment flow (number approached, eligible, consenting, and included) | Justification for site selection has been provided,incluing an a priori sample size calculation using G*Power 3.1 (minimum n=130; achieved n=134), and clarified our sampling approach in the revised methods section. | Line 125 to 134 |
| Questionnaire adaptation and validation-  The manuscript states that a questionnaire was adapted from Temtanakitpaisan et al., but the adaptation and validation process for the Ghanaian context is not described in sufficient detail. The authors should explain how the tool was translated, culturally adapted, and pretested, and provide more information on the pilot study beyond reporting a Cronbach’s alpha (e.g., what was assessed, what changes were made). | detailed information on the questionnaire adaptation process has been provided now, including specific examples of how items were modified for the Ghanaian context (e.g., splitting complex questions into clearer ones). pilot study description has been expanded to explain what was assessed (clarity and comprehensibility), how feedback from pilot participants was handled. Refer to the revised methods section | Line 143 to 165 |
| Statistical analysis and reporting-  The statistical methods are under described, and the analyses are almost descriptive. For transparency and rigor, the authors should (a) specify all statistical tests used, (b) report 95% confidence intervals for key estimates (means and proportions), and (c) where possible, explore simple associations (e.g., between socio-demographic factors, awareness, and practice of PFMEs). Clear denominators for all percentages in the tables are also needed. | continuous variables were expressed as means with standard deviations and 95% confidence intervals, while categorical variables were reported as frequencies, proportions, and 95% confidence intervals. Clear denominators have been included in all tables. chi-square analyses was attempted to explore associations, but violated assumptions (expected frequencies <5) prevented reliable results thus was not reported in this study | Line 167 to 171, line 185 to 129 |
| Results consistency and clarity- There are inconsistencies in the reporting of sample sizes, particularly between the 47 women reported as “aware” of PFMEs and the 24 participants included in parts of the beliefs analysis, which are not explained. The authors should clarify how many women contributed data to each section and explain any missing data. I also suggest simplifying and restructuring the large tables (especially Tables 3–5) and considering a simple flow diagram to show how the final analytical samples were derived. | The 24 mentioned earlier was an error and this has been rectified. The participants. The number of women contributing to data for each section has been stated now. Tables 4 to 5 has been restructured and simplified. | line 185 to 129; line 221 & 229 |
| Depth of discussion and interpretation-  The authors are encouraged to more fully contextualize their results by comparing with similar studies in other low and middle income as well as high income settings, to explore in more depth why awareness and positive beliefs do not translate into practice, and to discuss the practical implications for antenatal care services and physiotherapy provision. | The discussion section has been expanded to include comparisons with studies from both LMICs and HICs. Gaps between awareness, beliefs, and practice have been explored in depth, and practical implications for integrating PFME education into routine antenatal care, including healthcare provider training amongst others, have also been discussed | Line 244 to 340 |
| Limitations and reporting requirements-  The limitations section should be expanded to include the implications of convenience sampling from two facilities, relatively small sample size, reliance on self-reported data (including possible social desirability bias), and limited generalizability beyond the study sites. In addition, the manuscript should include a data availability statement, funding information, a competing interests declaration, and an author contributions statement in line with journal policies | The limitations section has been expanded to address convenience sampling from two facilities, sample size considerations, reliance on self-reported data with possible recall and social desirability bias, and limited generalizability beyond the study sites.  Reporting requirement such as  Competing interests declaration, Data availability statement, funding information and author contributions statement have been addressed using the PLOS ONE style templates as guide | Line 242 to 351 |
| Language and terminology-  The manuscript would benefit from careful language editing to correct grammatical errors and improve clarity (e.g., “aides vaginal birth” should be “aids vaginal birth”; “mean of gravida” should be “mean gravidity”). | This has been addressed | Line 37 and 187 |
